# One-Two Punch Therapy for the Treatment of T-Cell Malignancies Involving p53-Dependent Cellular Senescence

**DOI:** 10.1155/2021/5529518

**Published:** 2021-09-21

**Authors:** Yingjie Qing, Hui Li, Yunzi Zhao, Po Hu, Xiangyuan Wang, Xiaoxuan Yu, Mengyuan Zhu, Hongzheng Wang, Zhanyu Wang, Qinglong Guo, Hui Hui

**Affiliations:** ^1^State Key Laboratory of Natural Medicines, Jiangsu Key Laboratory of Carcinogenesis and Intervention, China Pharmaceutical University, 24 Tongjiaxiang, Nanjing 210009, China; ^2^School of Pharmacy, Nanjing University of Chinese Medicine, Nanjing, Jiangsu, 210023, China; ^3^Department of Pharmacology, School of Medicine & Holistic Integrative Medicine, Nanjing University of Chinese Medicine, Nanjing 210023, China

## Abstract

T-cell malignancies are still difficult to treat due to a paucity of plans that target critical dependencies. Drug-induced cellular senescence provides a permanent cell cycle arrest during tumorigenesis and cancer development, particularly when combined with senolytics to promote apoptosis of senescent cells, which is an innovation for cancer therapy. Here, our research found that wogonin, a well-known natural flavonoid compound, not only had a potential to inhibit cell growth and proliferation but also induced cellular senescence in T-cell malignancies with nonlethal concentration. Transcription activity of senescence-suppression human telomerase reverse transcriptase (*hTERT*) and oncogenic *C-MYC* was suppressed in wogonin-induced senescent cells, resulting in the inhibition of telomerase activity. We also substantiated the occurrence of DNA damage during the wogonin-induced aging process. Results showed that wogonin increased the activity of senescence-associated *β*-galactosidase (SA-*β*-Gal) and activated the DNA damage response pathway mediated by p53. In addition, we found the upregulated expression of BCL-2 in senescent T-cell malignancies because of the antiapoptotic properties of senescent cells. Following up this result, we identified a BCL-2 inhibitor Navitoclax (ABT-263), which was highly effective in decreasing cell viability and inducing apoptotic cell death in wogonin-induced senescent cells. Thus, the “one-two punch” approach increased the sensibility of T-cell malignancies with low expression of BCL-2 to Navitoclax. In conclusion, our research revealed that wogonin possesses potential antitumor effects based on senescence induction, offering a better insight into the development of novel therapeutic methods for T-cell malignancies.

## 1. Introduction

T-cell malignancies are a highly heterogeneous group of disorders [1, 2], which have been widely acknowledged to be typically aggressive and resistant to chemotherapy and have poor prognosis [3–5]. T-cell malignancies commonly consist of two categories, T-cell acute lymphoblastic leukemia (T-ALL) and T-cell non-Hodgkin lymphoma (T-NHL), both of which have similar clinical therapies and poor prognosis [6]. Due to the varied morphology of subtypes and being highly heterogeneous, the diagnosis and treatment of this disease have become a great challenge [7]. Although the standard first-line treatment of T-cell malignancies is a CHOP regimen (cyclophosphamide, doxorubicin, vincristine, and prednisone), which is still the representative chemotherapy [8], most patients still have a poor prognosis with an ORR of 65.8% because of rapid relapse [8]. Relapse and cytotoxicity always become an unavoidable problem for several patients. And just a few of patients who achieve remission are suitable for hematopoietic stem cell transplantation [9, 10]. Thus, it is urgent to find more effective therapy options for patients who fail to respond to chemotherapy or relapse after chemotherapy.

Cellular senescence is a complex stress response accompanied by permanent cell cycle arrest and inhibition of proliferation, which is induced by DNA damage, telomere shortening, ionizing radiation, or oncogene [11, 12]. The typical characteristics of senescent cells are large and flat morphology and positive expression of senescence-associated *β*-galactosidase. Moreover, senescence-associated heterochromatin foci (SAHF) are also a biomarker of senescent cells, which are formed by nuclei as the heterochromatin structures [13]. In hematologic malignancies, cells abnormally overexpress human telomerase reverse transcriptase and decrease the expression of senescence-promoting genes, which contribute to immortalization of cancer cells [14]. Previous reports have shown that *hTERT* also exerts antisenescent effect through telomerase- and telomere length-independent mechanisms [15]. As a novel therapeutic concept, the use of drugs which can accumulate DNA damage and trigger cellular senescence in cancer cells can provide a new insight into treating hematologic malignancies. However, induced cellular senescence alone cannot achieve the ideal therapeutic effect. Many chemotherapy drugs can induce cellular senescence and alert the tumor microenvironment, which is a double-edged sword for cancer therapy [11, 16]. Although senescent cells induced by agents had vulnerabilities such as persistent growth inhibition, persisting existence of senescent cells can promote cancer progression and invasion in B-cell lymphoma and T-cell acute lymphoblastic leukemia [17]. Thus, exploiting vulnerabilities and removing senescent cells are important for the second step of senescence-focused cancer therapies [18–20]. As the characteristic of resistance to apoptosis in senescent cells, senolytics are identified to eliminate aging cells including quercetin/dasatinib, pan-BCL inhibitors through the inhibition of the BCL-2 family of proteins (BCL-2, BCL-xL, and BCL-w) [21, 22]. Therefore, a one-two punch cancer therapy may be regarded as a potentially therapeutic strategy [20].

Nowadays, DNA-damaging drugs are widely used in cancer therapy, which not only induce cell death but also activate cellular senescence [23]. DNA damage response (DDR) plays a core role in senescence. The biomarker of DDR is the activation of H2A.X, which depends on the phosphorylation at Ser139 (*γ*-H2AX). As a main factor of DDR, p53 acts as a protagonist during the process of aging and tumor suppression [24]. A previous study reported that in adult acute lymphoblastic leukemia, the alterations of *TP53*, the most important tumor suppressor gene, including deletions, sequence mutations, and defect in its expression, result in an adverse prognosis [25]. As the activation of DNA damage, p53 is activated through phosphorylation on serine-15 and serine-20 by ataxia-telangiectasia mutated (ATM) or ataxia-telangiectasia Rad3-related (ATR) and the checkpoint kinase 2 (CHK2), respectively [26, 27]. And then, the interaction of MDM2 and p53 is impaired, promoting the accumulation and stabilization of p53 in response to DNA damage activation [28]. After activation of p53, the downstream effectors like p21 regulate different cell responses such as cell cycle arrest and senescence.

Wogonin (5,7-dihydroxy-8-methoxy-2-phenyl-4h-1-benzopyran-4-one) is a type of flavonoid extracted from *S. baicalensis* Georgi, which has prominent biological activities including anti-inflammatory, antiviral, and anticancer activities [29, 30]. However, cytostatic concentrations of wogonin have other characteristics such as inducing cellular senescence, which can combine with senolytics as a new therapeutic regimen. Here, we first investigated wogonin-induced cellular senescence and found the suppression of telomerase activity in wogonin-treated senescent cells. Moreover, DNA damage response mediated by p53 was involved in cellular senescence induced by wogonin. Finally, we also clarified that the combination sequential treatment of wogonin and Navitoclax, a BCL-2 inhibitor, could obtain better effect and become a novel strategy in T-cell malignancies.

## 2. Materials and Methods

### 2.1. Compounds and Reagents

Wogonin (purity ≥ 99%) is isolated from *S. baicalensis* Georgi according to previously reported protocols [31]. Wogonin was dissolved in dimethyl sulfoxide (DMSO) as a stock solution (100 mM) and stored at -80°C. The solution was freshly diluted with basal medium to designated concentrations, and the final concentration of DMSO will not be over 0.1%. Cells treated with the highest concentration of DMSO were used as the control in corresponding experiments. Navitoclax (CSN12932) was purchased from CSNpharm (Chicago, USA). Navitoclax powder was dissolved with DMSO to 10 mM and stored at -20°C. Primary antibodies against *β*-actin, GAPDH, trimethyl-histone H3-K9, BCL-2, hTERT, c-Myc, caspase-3, active caspase-3, p27, Bax, CHK2, cyclin D1, cyclin E1, CDK4, CDK6, HRP goat anti-mouse IgG (H+L), and HRP goat anti-rabbit IgG (H+L) were obtained from ABclonal Technology (Wuhan, China). Phospho-CHK2 (T68), phospho-p53 (Ser15), p21, and phospho-histone H2A.X (*γ*-H2A.X) were obtained from Cell Signaling Technology (Danvers, MA, USA). p53, p16, BCL-xL, Bim, and PARP-1 were obtained from Proteintech (CA, USA).

### 2.2. Cell Culture

The human T-ALL cell line (Jurkat cells), human T-cell lymphoma cell lines (Hut-102 and Hut-78 cells), and HEK293T cells were purchased from the Cell Bank of Shanghai Institute of Biochemistry & Cell Biology. All the cells were expanded and stored in liquid nitrogen upon receipt, and each aliquot was passaged for fewer than 25-30 times in our laboratory. Jurkat, Hut-102, and Hut-78 cells were cultured in RPMI-1640 medium (Gibco, Carlsbad, USA). HEK293T cells were cultured in DMEM medium (Gibco, Carlsbad, USA). Medium was supplemented with 10% FBS (Thermo Fisher Scientific, Waltham, MA), 100 U/mL of penicillin, and 100 U/mL of streptomycin in a humidified environment (Thermo Fisher Scientific) with 5% CO_2_ at 37°C.

### 2.3. Cell Viability Assay

Cell proliferation of cells was detected using Cell Counting Kit-8 (KeyGen Biotech, Nanjing, China) according to the manufacturer's instruction. Briefly, cells (1 × 10^4^ cells/well) were seeded into a 96-well plate in 100 *μ*L medium and treated with wogonin at different concentrations for 24 h. Each group consisted of six parallel wells. Then, 10 *μ*L of CCK8 solution was added to each well, and cells were incubated at 37°C for 1–4 h. The absorbance was measured at 450 nm using an Automated Microplate Reader ELx800 (BioTek). The cell survival rate (%) was calculated using the following equation: survival rate (%) = (OD_treated_ − OD_blank_)/(OD_standard_ − OD_blank_) × 100%.Inhibition rate (%) = 100% − survival rate (%).

### 2.4. Quantitative Real-Time RT-qPCR

RT-qPCR was performed according to the manufacturer's instructions [32].

The primer sequences were as follows:

*GAPDH*

Forward 5′-GCAGGGGGGAGCCAAAAGGG-3′

Reverse 5′-TGCCAGCCCCAGCGTCAAAG-3′

*P16*

Forward 5′-GATCCAGGTGGGTAGAAGGTC-3′

Reverse 5′-CCCCTGCAAACTTCGTCCT-3′

*P21*

Forward 5′-TGTCCGTCAGAACCCATGC-3′

Reverse 5′-AAAGTCGAAGTTCCATCGCTC-3′

*P27*

Forward 5′-AACGTGCGAGTGTCTAACGG-3′

Reverse 5′-CCCTCTAGGGGTTTGTGATTCT-3′

*C-MYC*

Forward 5′-GGCTCCTGGCAAAAGGTCA-3′

Reverse 5′-CTGCGTAGTTGTGCTGATGT-3′

*hTERT*

Forward 5′-TATGTCACGGAGACCACGTT-3′

Reverse 5′-GTGCTGTCTGATTCCAATGC-3′

### 2.5. Flow Cytometric Analysis of Apoptosis

Apoptosis was evaluated using an Annexin V-FITC/PI Apoptosis Detection Kit (KeyGen Biotech, Nanjing, China) according to the manufacturer's instruction. Cellular fluorescence was performed by flow cytometric analysis using a FACSCalibur flow cytometer (Becton Dickinson Biosciences, San Jose, CA, USA). Our data was analyzed by using FlowJo software.

### 2.6. Cell Cycle Analysis

Cells were collected and washed with PBS and then fixed with 70% ethanol for 2 h at 4°C. After fixation, the cells were washed with PBS buffer and stained with PI for 30 min. The fluorescence was detected by flow cytometry. The cell cycle was analyzed and quantified using a BD FACSCalibur flow cytometer (Becton Dickinson, San Jose, CA). The percentage of cells in G0/G1, S, and G2/M phases of the cell cycle was determined by the PI fluorescence signal peak versus the integral which was analyzed by using ModFit LT 4.0 software (Becton Dickinson, San Jose, CA, USA).

### 2.7. CFSE Assay

CFSE-labeled cells (CellTrace CFSE Proliferation Kit, Invitrogen) were cultured with wogonin for the indicated time, cells were harvested, and cell proliferation capacity was determined by using a flow cytometer.

### 2.8. Cell Staining of Senescence-Associated *β*-Galactosidase (SA-*β*-Gal)

SA-*β*-Gal activity was measured by using a *β*-Galactosidase Staining Kit (Yeasen, Shanghai, China) according to the standard protocol. The stained cells were observed and photographed under the microscope (Carl Zeiss, Germany). Cells were considered positive when the cytoplasm was stained with SA-*β*-Gal. Total numbers of cells and SA-*β*-Gal-positive cells were counted for five fields per well. The ratios of SA-*β*-Gal-positive cells were calculated compared to the total cell numbers per area.

### 2.9. Flow Cytometric Detection of SA-*β*-Gal

For flow cytometry-based detection of senescence, the fluorogenic substrate 5-dodecanoylaminofluorescein di-beta-d-galactopyranoside (C_12_FDG) was used as described previously [33]. According to the manufacturer's instructions, cells were incubated with 33 *μ*M C_12_FDG (ImaGene Green C_12_FDG lacZ Gene Expression Kit) at 37°C for 30 min. Then, cells were washed with PBS and measured immediately with a FACSCalibur flow cytometer (Becton Dickinson Biosciences, San Jose, CA, USA). Data were analyzed using FlowJo software. SA-*β*-Gal activity was estimated using the relative mean fluorescence intensity of the cell population.

### 2.10. Western Blotting Analysis

Whole cell proteins were collected after the treatment with wogonin and were extracted by using RIPA buffer containing protease/phosphatase inhibitors on ice for 50 min. Then, cell lysates were clarified by centrifugation at 14,000 rpm (5430R; Eppendorf, Hamburg, Germany) for 25 min at 4°C. Concentrations of proteins were measured using the BCA Protein Assay Kit (Thermo Fisher Scientific, USA). Protein extracts were equally loaded on 8%-12% SDS-polyacrylamide gels and then transferred onto the PVDF membranes (Millipore, Boston, MA, USA). Membranes were blocked with 3% BSA in PBS at room temperature for 1 h and then incubated with primary antibodies overnight at 4°C. After that, the membrane was incubated with an HRP goat anti-rabbit IgG (H+L) or HRP goat anti-mouse IgG (H+L) secondary antibody for 1 h at room temperature. Chemiluminescence detection was performed by using ECL reagents (Thermo Fisher Scientific, USA) upon using the AI600 imaging system (GE Healthcare, Pittsburgh, PA, USA).

### 2.11. Immunofluorescence

Cells were collected and seeded onto glass coverslips. Then, the cells were fixed in the ice-cold methanol for 10 min and incubated with 0.15% (*v*/*v*) Triton X-100 for 20 min. After the cells were blocked with 3% BSA at room temperature for 1 h, the cells were incubated with primary antibodies overnight at 4°C. Following this, the cells were stained with an Alexa Fluor 488-conjugated goat anti-rabbit IgG secondary antibody (Becton Dickinson) for 1 h and DAPI (Beyotime Biotechnology, Shanghai, China) for 5 min. Cells were observed under a confocal laser scanning microscope (FV1000; Olympus, Tokyo, Japan).

### 2.12. RNA Interference

For RNA interference by lentiviral vectors, *P53* shRNA constructs and a negative control construct created in the same vector system (PLV3ltr-puro-U6) were purchased from Corues Biotechnology. A *P53* shRNA construct and a negative control were transfected into HEK293T cells together with the Lentiviral Mix and HG Transgene™ Reagent according to the manufacturer's instruction of the Lentiviral Packaging Kit (Yeasen, 41102ES20) for 48 h. After this, the virus supernatant was harvested. Then, the virus supernatant infected target cells. 2 *μ*g/mL puromycin was added to screen for expressing shRNA construct cells.

### 2.13. Statistical Analysis

All results were expressed as the mean ± standard error of the mean (SEM). The data shown were obtained from triplicate independent parallel experiments. Statistical analyses were performed by using one-way analysis of variance (ANOVA), post hoc intergroup comparisons, and Tukey's test. Two-tailed Student's *t*-test was used to determine differences between groups. ^∗^*p* < 0.05 was considered statistically significant, ^∗∗^*p* < 0.01 very significant, and ^∗∗∗^*p* < 0.001 highly significant. Statistical analyses were performed using GraphPad Prism 6.0 Software (GraphPad, San Diego, CA).

## 3. Results

### 3.1. Wogonin Induced Growth Inhibition but Not Apoptosis in Hut-102 and Jurkat Cells

**Figure 1:**
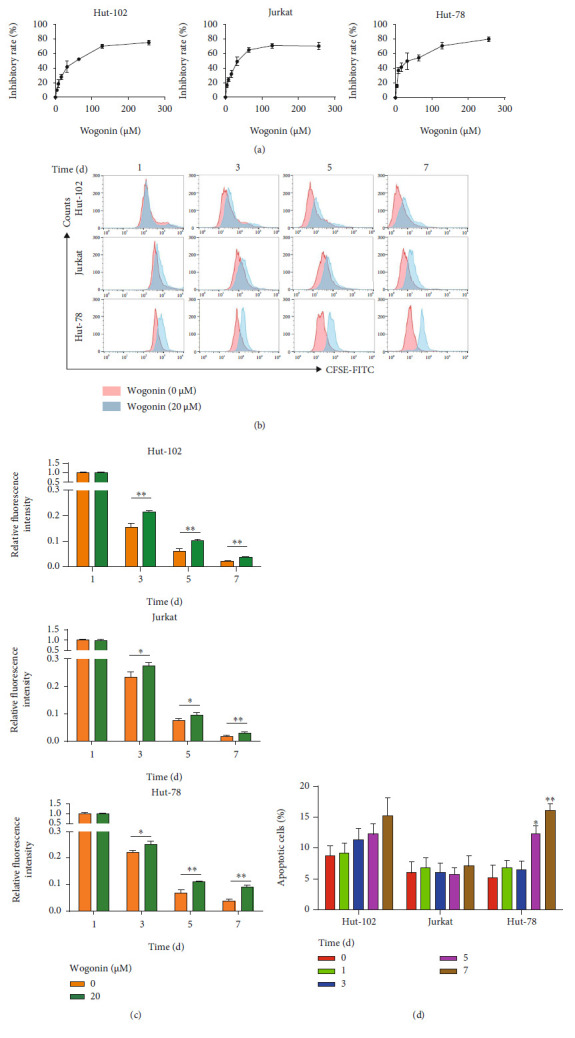
Wogonin induced growth inhibition but not apoptosis in Hut-102 and Jurkat cells. (a) Hut-102, Jurkat, and Hut-78 cells were exposed to wogonin at indicated concentrations (0-256 *μ*M), respectively. The inhibitory rate of cell viability was measured by the CCK8 assay after 24 h. (b) Hut-102, Jurkat, and Hut-78 cells were treated with or without 20 *μ*M wogonin; then, the cells were labeled with CFSE for 1, 3, 5, and 7 d, respectively. Results of CFSE expression were analyzed by flow cytometry. (c) Quantification of CFSE expression. Ordinate represents relative changes of fluorescence intensity (GEOmean). Significant difference represents the fluorescent intensity between the control group and wogonin group in the same day. Columns represent the mean from three parallel experiments (mean ± SEM). ^∗^*p* < 0.05, ^∗∗^*p* < 0.01, compared with the control group. (d) Hut-102, Jurkat, and Hut-78 cells were treated with 20 *μ*M wogonin for the indicated times (0, 1, 3, 5, and 7 d). Then, the cell apoptosis rates were analyzed via Annexin V/PI staining by flow cytometry. Columns represent the mean from three parallel experiments (mean ± SEM). ^∗^*p* < 0.05, ^∗∗^*p* < 0.01, compared with the control group.

In order to investigate the growth suppression effects of wogonin on T-cell malignancy cells, the growth inhibition rates of cells were detected by the CCK8 assay firstly. We performed our studies using T-NHL cell lines (Hut-102 and Hut-78) and the T-ALL cell line (Jurkat). Cells were treated with various concentrations of wogonin (0–256 *μ*M) for 24 h. As shown in Figure 1(a), wogonin inhibited cell proliferation in a concentration-dependent manner. To investigate whether low concentration of wogonin still has proliferation inhibition on T-cell malignancies for a long-time exposure, we chose 20 *μ*M as the treatment concentration. 20 *μ*M wogonin inhibited cell proliferation in T-cell malignancies notably, as the decreased fluorescence intensity in CFSE-labeled cells was prevented by wogonin (Figures 1(b) and 1(c)). However, 20 *μ*M wogonin did not induce significant apoptosis in Hut-102 and Jurkat cells after treatment for 1, 3, 5, and 7 d, but in Hut-78 cells, apoptosis rates showed a slight increase (Figure 1(d)). It is concluded that wogonin suppressed growth and proliferation of human T-cell malignancy cells.

### 3.2. Wogonin Induced Cellular Senescence in Hut-102 and Jurkat Cells but Not Hut-78 Cells

**Figure 2:**
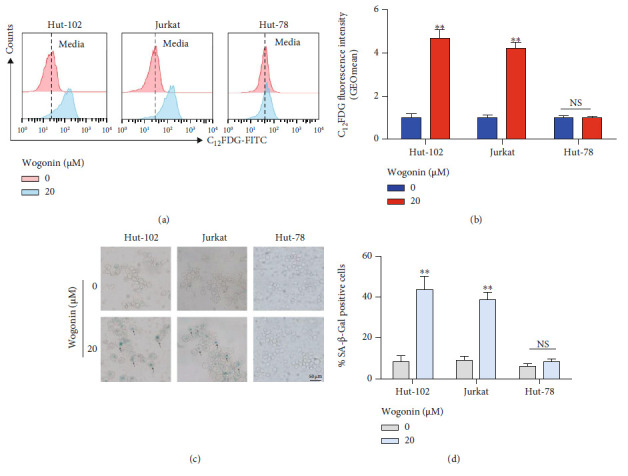
Wogonin induced cellular senescence in Hut-102 and Jurkat cells but not Hut-78 cells. (a) Cells (Hut-102, Jurkat, and Hut-78 cells) were treated with or without 20 *μ*M wogonin for 5 d. SA-*β*-Gal activity was measured by C_12_FDG fluorogenic substrate of SA-*β*-galactosidase using flow cytometry. Representative peak graphs from one of the three independent experiments, illustrating C_12_FDG fluorescence. (b) Quantification of SA-*β*-Gal activity measured by C_12_FDG fluorescence. The dark blue histogram represents control cells, and the red histogram represents cells that were cultured in the presence of 20 *μ*M wogonin. Columns represent the mean from three parallel experiments (mean ± SEM). ^∗^*p* < 0.05, ^∗∗^*p* < 0.01, compared with the control group. (c) SA-*β*-Gal activity was measured by SA-*β*-Gal staining in cells (Hut-102, Jurkat, and Hut-78 cells) treated with or without 20 *μ*M wogonin for 5 d. Representative images from one of the three independent experiments. Representative pictures are shown (magnification, ×200). (d) Quantification of SA-*β*-Gal activity measured by cell staining. The grey histogram represents control cells, and the blue histogram represents cells that were cultured in the presence of 20 *μ*M wogonin. Columns represent the mean from three parallel experiments (mean ± SEM). ^∗^*p* < 0.05, ^∗∗^*p* < 0.01, compared with the control group.

A previous study has confirmed that small-molecule compounds can induce the cellular senescence in a few of cases [34]. It is a viable therapeutic option named one-two punch therapy to induce senescence of cancer cells and then remove them [19]. In order to validate the one-two punch therapy for the treatment of T-cell malignancies, firstly we identified whether wogonin could induce cellular senescence in these T-cell malignancy cells. C_12_FDG and senescence-associated *β*-galactosidase staining test were adopted to assess the cellular senescence in Hut-102, Jurkat, and Hut-78 cells treated with 20 *μ*M wogonin for 5 d. Higher SA-*β*-Gal activity measured by C_12_FDG using flow cytometry was observed in wogonin-treated Hut-102 and Jurkat cells (Figures 2(a) and 2(b)). Moreover, more positive *β*-galactosidase-stained cells were found in wogonin-treated Hut-102 and Jurkat cells compared with control cells (Figures 2(c) and 2(d)). Morphologically, the senescent cells were enlarged and flattened compared with control cells (Figures 2(c) and 2(d)). Interestingly, cellular senescence was not observed in Hut-78 cells after wogonin treatment for 5 d (Figures 2(a) – 2(d)). Overall, these results showed that low concentration of wogonin (20 *μ*M) could induce cellular senescence in Hut-102 and Jurkat cells.

### 3.3. Wogonin-Induced Cell Cycle Arrest Was Accompanied by Cellular Senescence

**Figure 3:**
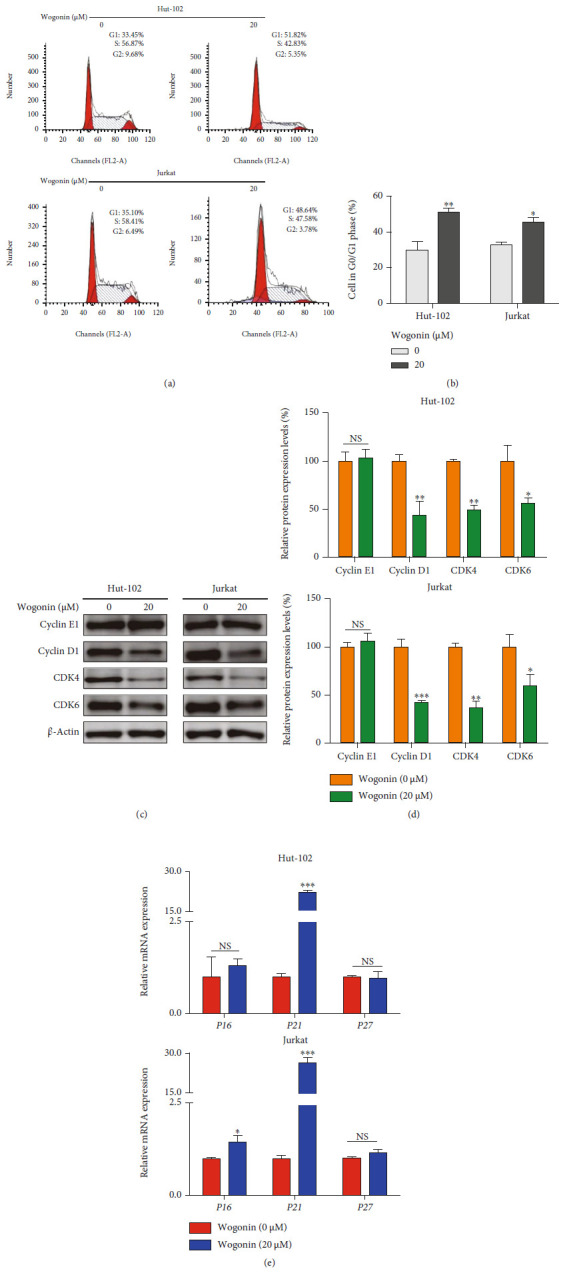
Wogonin-induced cell cycle arrest was accompanied by cellular senescence. (a) Hut-102 and Jurkat cells were treated with or without 20 *μ*M wogonin for 5 d. Representative cell cycle was performed by PI staining and analyzed by flow cytometry. (b) The percentages of cells in the G_0_/G_1_ phases of the cell cycle following 20 *μ*M wogonin treatment for 5 d are shown. Columns represent the mean from three parallel experiments (mean ± SEM). ^∗^*p* < 0.05, ^∗∗^*p* < 0.01, compared with the control group. (c) Hut-102 and Jurkat cells were treated with or without 20 *μ*M wogonin for 5 d. Western blot analyses of cell cycle regulatory proteins cyclin E1, cyclin D1, CDK4, and CDK6. *β*-Actin was used as loading controls. (d) Relative protein expression levels of cyclin D1, cyclin E1, CDK4, and CDK6 were determined. Columns represent the mean from three parallel experiments (mean ± SEM). ^∗^*p* < 0.05, ^∗∗^*p* < 0.01, compared with the control group. (e) The relative mRNA levels of *P16*, *P21*, and *P27* in control and wogonin-treated Hut-102 and Jurkat cells. Columns represent the mean from three parallel experiments (mean ± SEM). ^∗^*p* < 0.05, ^∗∗^*p* < 0.01, and ^∗∗∗^*p* < 0.001, compared with the control group.

Cell cycle blockage is a well-known feature of cellular senescence, which contributes to permanent growth arrest in senescent cells [35, 36]. Thus, the effects of wogonin on cell cycle progression were measured in Hut-102 and Jurkat cells treated with 20 *μ*M wogonin for 5 d. Results showed that the proportion of cells increased in the G_1_ phase and decreased in the S phase compared to the untreated cells (Figures 3(a) and 3(b)). The CDK4/6-cyclin D1 complex plays a key role in promoting transition of the G_1_ to S phrase, and the CDK2-cyclin E1 complex, the downstream of the CDK4/6-cyclin D1 complex, promotes cells to enter the S phrase through the restriction point [37]. Western blots indicated that wogonin decreased the expression of cyclin D1, CDK4, and CDK6, while the cyclin E1 expression was stable (Figures 3(c) and 3(d)). Furthermore, the increased expression of p16,p21, and p27 may contribute to cell cycle blockage in senescent cells [38, 39]. RT-PCR results showed that wogonin increased the mRNA level of *P21* by over 20-fold in senescent Hut-102 and Jurkat cells, while the mRNA level of *P27* did not change in those two cell lines (Figure 3(e)). The mRNA level of *P16* was slightly increased in Jurkat cells but not in Hut-102 cells (Figure 3(e)). All these data demonstrated that wogonin arrested the cell cycle at the checkpoint of G_0_/G_1_, which was correlated with cellular senescence in T-cell malignancy cell lines including Hut-102 and Jurkat.

### 3.4. Wogonin Suppressed Telomerase Activity by Inhibition of hTERT and c-Myc in Wogonin-Induced Senescent Cells

**Figure 4:**
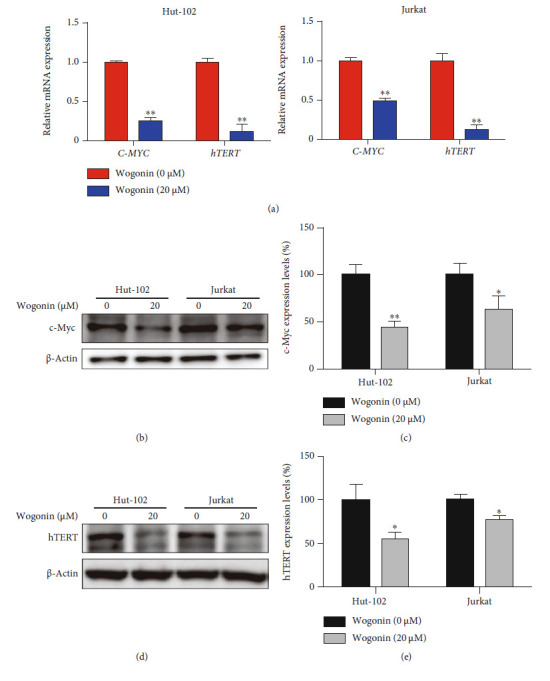
Wogonin suppressed telomerase activity by inhibition of hTERT and c-Myc in wogonin-induced senescent cells. (a) Hut-102 and Jurkat cells were treated with or without 20 *μ*M wogonin for 5 d. The relative mRNA level of *hTERT* and *C-MYC* was measured by RT-PCR. Columns represent the mean from three parallel experiments (mean ± SEM). ^∗^*p* < 0.05, ^∗∗^*p* < 0.01, compared with the control group. (b) Hut-102 and Jurkat cells were treated with or without 20 *μ*M wogonin for 5 d. The expression of c-Myc protein was performed by western blot. *β*-Actin was used as loading controls. (c) Relative protein expression level of c-Myc. Columns represent the mean from three parallel experiments (mean ± SEM). ^∗^*p* < 0.05, ^∗∗^*p* < 0.01, compared with the control group. (d) Hut-102 and Jurkat cells were treated with or without 20 *μ*M wogonin for 5 d. The expression of hTERT protein was performed by western blot. *β*-Actin was used as loading controls. (e) Relative protein expression level of hTERT. Columns represent the mean from three parallel experiments (mean ± SEM). ^∗^*p* < 0.05, ^∗∗^*p* < 0.01, compared with the control group.

Telomere length affects cell life, and when the telomere shortens to a certain extent, cell senescence occurs [40]. Previous studies have shown that telomerase activity affects telomere length [41]. As a main component of telomerase, human telomerase reverse transcriptase is overexpressed in many malignant tumors including hematologic malignancies [42, 43]. c-Myc, a transcription factor, binds to the promoter region of *hTERT* and drives overexpression of *hTERT* in cancer cells, resulting in the activation of telomerase [44]. In order to verify whether wogonin induced cellular senescence involved in inhibiting telomerase activity, we first detect the mRNA levels of *C-MYC* and *hTERT*. Results showed that the mRNA expression of *C-MYC* and *hTERT* were decreased in Hut-102 and Jurkat cells after treatment with 20 *μ*M wogonin for 5 d (Figure 4(a)). By the way, we also investigate the protein expression of c-Myc and hTERT using western blot, and results showed that wogonin decreased the expression of c-Myc and hTERT in Hut-102 and Jurkat cells after treatment for 5 d (Figures 4(b) – 4(e)). Thus, we concluded that wogonin could inhibit telomerase activity in senescent cells.

### 3.5. Wogonin Induced Senescence-Associated Heterochromatin Foci in Senescent Cells

**Figure 5:**
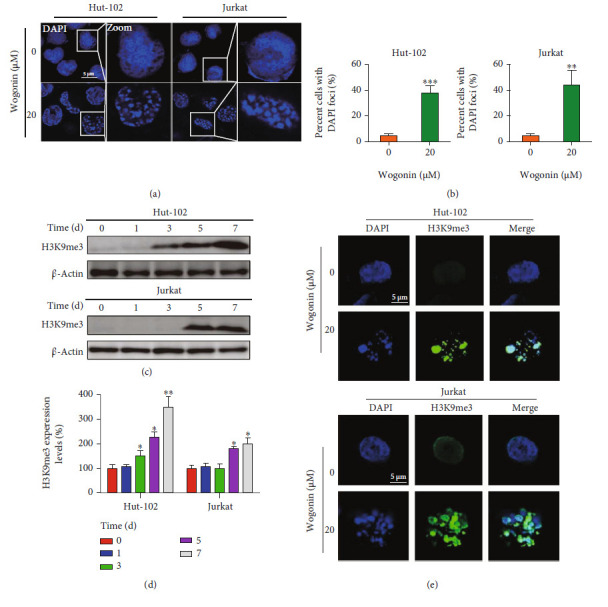
Wogonin induced senescence-associated heterochromatin foci in senescent cells. (a) Hut-102 and Jurkat cells were treated with or without 20 *μ*M wogonin for 5 d; then, the cells were fixed and permeabilized, and nuclei were stained with DAPI (blue). SAHF formed in wogonin-treated Hut-102 and Jurkat cells. Representative confocal images for wogonin-induced SAHF formation in cells from confocal laser scanning microscopy are shown (original magnification ×1000; immersion objective ×100/×100 with immersion oil type). Images are representative of three independent experiments. (b) The percentage of positive cells with the DNA agglutination points (DAPI foci) was counted (with 20 cells counted per field). Columns represent the mean from three parallel experiments (mean ± SEM). ^∗∗^*p* < 0.01, ^∗∗∗^*p* < 0.001, compared with the control group. (c) Hut-102 and Jurkat cells were treated with or without 20 *μ*M wogonin for 5 d. The expression of the SAHF marker protein H3K9me3 in control and wogonin-treated Hut-102 and Jurkat cells was performed by western blot. *β*-Actin was used as loading controls. (d) Relative protein expression level of H3K9me3 was determined. Columns represent the mean from three parallel experiments (mean ± SEM). ^∗^*p* < 0.05, ^∗∗^*p* < 0.01, compared with the control group. (e) Hut-102 and Jurkat cells were treated with or without 20 *μ*M wogonin for 5 d; then, the cells were fixed, permeabilized, and stained with an antibody against H3K9me3 (green), while nuclei were stained with DAPI (blue). Immunofluorescent images showed the distribution of H3K9me3 in Hut-102 and Jurkat cells (original magnification ×1000; immersion objective ×100/×100 with immersion oil type). Images are representative of three independent experiments.

Cell senescence is accompanied by the change of chromosome structure, especially the phenomenon of heterochromatin foci, which is the biological marker of cell senescence [13, 45]. In order to determine whether wogonin induced SAHF in senescent cells, Hut-102 and Jurkat cells were exposed to 20 *μ*M wogonin for 5 d and formation of DNA agglutination points appeared (Figure 5(a)). The ratios of agglutination point-positive cells were 38.00% ± 5.10% and 44.22 ± 11.09% in wogonin-treated Hut-102 and Jurkat cells, respectively (Figure 5(b)). Next, we verified the molecular marker of SAHF named H3K9me3 by western blots and under a laser scanning confocal microscope. Results of western blots showed that the expression of H3K9me3 was upregulated after the treatment with wogonin for 3, 5, and 7 d in Hut-102 cells; meanwhile, H3K9me3 was also upregulated after the treatment with wogonin for 5 and 7 d in Jurkat cells (Figures 5(c) and 5(d)). Immunofluorescence analysis showed that the fluorescence intensity of H3K9me3 increased and colocated with the agglutination point in Hut-102 and Jurkat cells (Figure 5(e)).

### 3.6. Wogonin Induced Cellular Senescence through p53-Mediated DNA Damage Response

**Figure 6:**
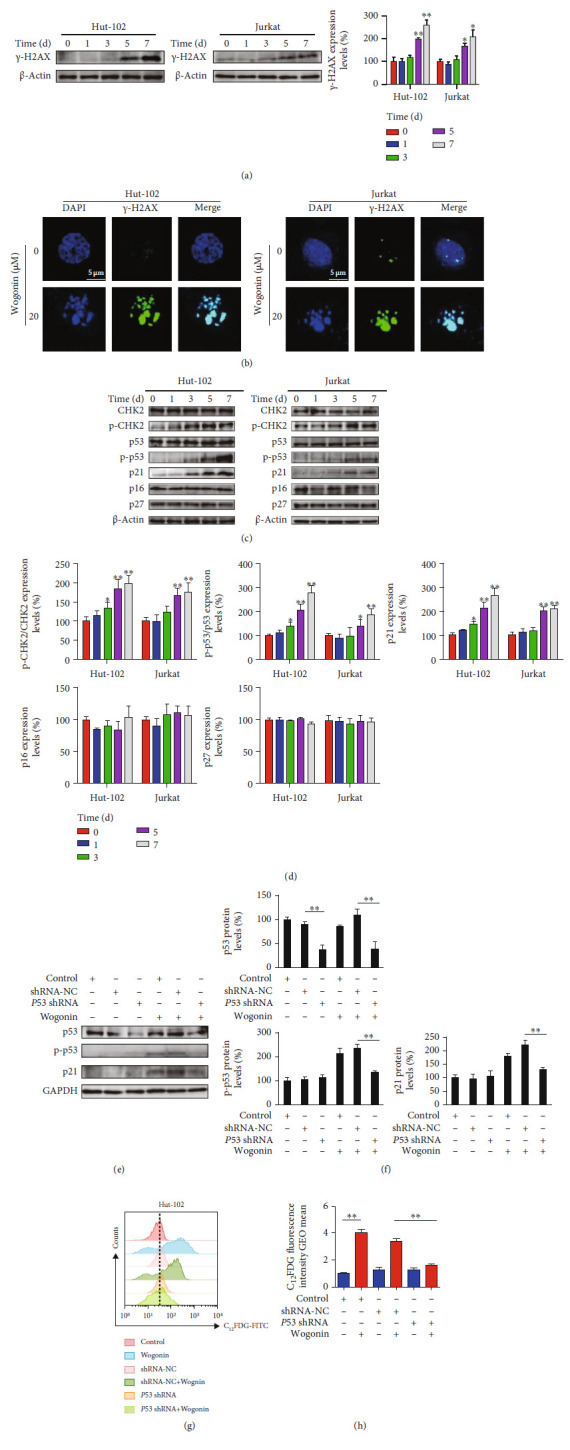
Wogonin induced cellular senescence through p53-mediated DNA damage response. (a) Hut-102 and Jurkat cells were treated with or without 20 *μ*M wogonin for the indicated time periods, and western blot was used to detect the expression change of the DNA damage marker protein *γ*-H2AX. *β*-Actin was used as loading controls. Relative protein expression level of *γ*-H2AX was determined. Columns represent the mean from three parallel experiments (mean ± SEM). ^∗^*p* < 0.05, ^∗∗^*p* < 0.01, compared with the control group. (b) Hut-102 and Jurkat cells were treated with or without 20 *μ*M wogonin for 5 d; then, the cells were fixed, permeabilized, and stained with an antibody against *γ*-H2AX (green), while nuclei were stained with DAPI (blue). Immunofluorescent images showed the distribution of *γ*-H2AX in Hut-102 and Jurkat cells (original magnification ×1000; immersion objective ×100/×100 with immersion oil type). Images are representative of three independent experiments. (c) Hut-102 and Jurkat cells were treated with or without 20 *μ*M wogonin for the indicated time periods (0, 1, 3, 5, and 7 d), and western blot was performed to detect the expression changes of CHK2, p-CHK2 (T68), p53, p-p53 (Ser15), p21, p16, and p27. *β*-Actin was used as loading controls. (d) Relative protein expression levels of p-CHK2/CHK2, p-p53/p53, p21, p16, and p27 were determined. Columns represent the mean from three parallel experiments (mean ± SEM). ^∗^*p* < 0.05, ^∗∗^*p* < 0.01, compared with the control group. (e) The protein expression levels of p53, p-p53 (Ser15), and p21 were detected by western blot. *P53* shRNA-transfected Hut-102, shRNA-NC, and control Hut-102 cells were treated with or without 20 *μ*M wogonin for 5 d. GAPDH was used as loading controls. (f) Relative protein expression levels of p53, p-p53 (Ser15), and p21. Columns represent the mean from three parallel experiments (mean ± SEM). ^∗^*p* < 0.05, ^∗∗^*p* < 0.01, compared with the control group. (g) Hut-102 cells were transfected with *P53* shRNA and shRNA-NC by a lentiviral vector. *P53* shRNA-transfected Hut-102, shRNA-NC, and control Hut-102 cells were treated with or without 20 *μ*M wogonin for 5 d. The activity of SA-*β*-Gal was measured by flow cytometry (cells were stained with C_12_FDG). (h) Relative SA-*β*-Gal activity was assessed by using the GEOmean of groups, which represented C_12_FDG fluorescence intensity. Columns represent the mean from three parallel experiments (mean ± SEM). ^∗^*p* < 0.05, ^∗∗^*p* < 0.01, compared with the NC group.

Many studies have shown that anticancer agent-induced cellular senescence is mediated by the DNA damage pathway [24, 46]. To figure out the mechanism of cellular senescence induced by wogonin, we first performed western blot experiments to detect the protein expression of the DNA damage marker protein *γ*-H2AX in Hut-102 and Jurkat cells. We found that the *γ*-H2AX expression increased obviously after treatment with 20 *μ*M wogonin for 5 d and 7 d (Figure 6(a)), while in Hut-78 cells, the level of *γ*-H2AX was increased slightly with the treatment with 20 *μ*M wogonin for 7 d (Figure S1a). In line with that, results of the immunofluorescence experiment showed that the fluorescence intensity of *γ*-H2AX was enhanced and colocalized with the nucleus after treatment with 20 *μ*M wogonin in three T-cell malignancies (Figure 6(b), Figure S1b). The enhanced expression of *γ*-H2AX proved the wogonin-induced DNA damage response. We then analyzed the activation level of the CHK2/p53/p21 axis, which is involved in DNA damage response [47]. Western blot indicated that the level of p-CHK2 (Thr68) was gradually upregulated in wogonin-treated Hut-102 and Jurkat cells in a time-dependent manner, as well as p-p53 (Ser15) and p21. However, the expression of CHK2, p53 and p16, and p27 was stable in Hut-102 and Jurkat cells after treatment with wogonin (Figures 6(c) and 6(d)). But in Hut-78 cells, a cell line lacking p53 expression resulted from a homozygous point mutation at codon 196 (CGA-TGA) [48] and the level of CHK2 and p-CHK2 (Thr68) was increased after treatment with wogonin for 7 d; meanwhile, p21 was slightly increased and p16 and p27 expression was not changed (Figure S1c, d). However, the change degree of p21 in Hut-78 cells is generally slighter than that in Hut-102 and Jurkat cells (Figure 6(d), Figure S1d).

To further investigate whether wogonin-induced senescence depended on the functionally intact p53, we performed our studies using lentiviral-transfected *P53* shRNA and negative control (NC) in wt-p53 Hut-102 cells. Transfected efficiency of *P53* shRNA is shown in Figures 6(e) and 6(f). Flow cytometric analysis showed that the activity of SA-*β*-Gal alleviated in cells transfected with *P53* shRNA compared with the negative control group after wogonin treatment (Figures 6(g) and 6(h)). Moreover, western blot showed that wogonin-induced expression of p-p53 (Ser15) and p21 was inhibited by *P53* knockdown (Figures 6(e) and 6(f)). As a result, DNA damage response is involved in wogonin-induced cellular senescence, and the participation of p53 is critical for the cellular senescence induced by wogonin.

### 3.7. Wogonin-Induced Cellular Senescence Sensitized Navitoclax to Kill T-Cell malignancy cells.

**Figure 7:**
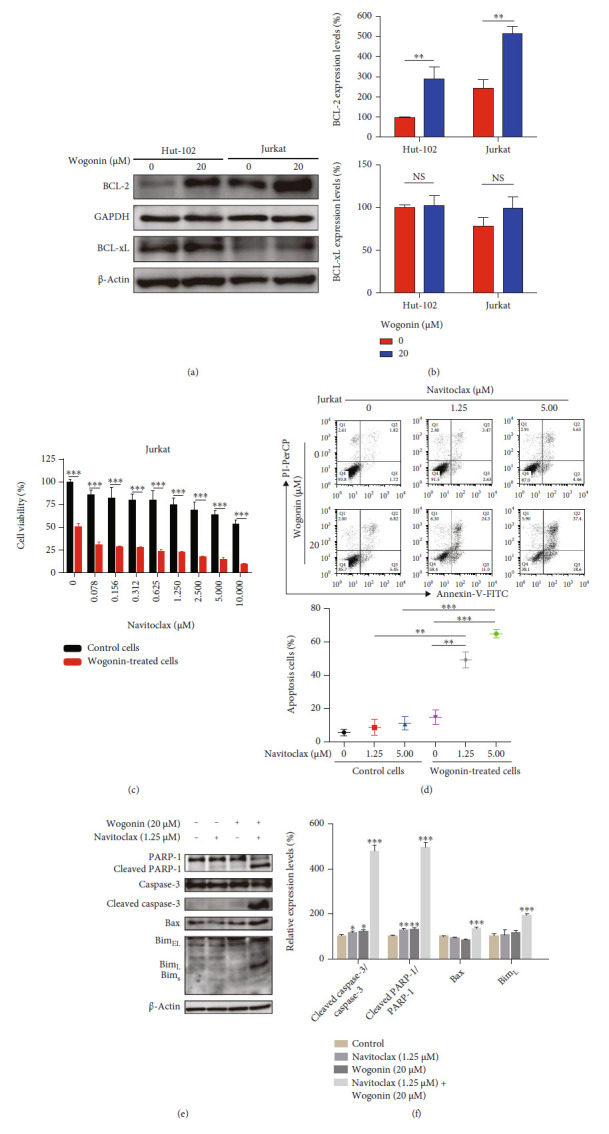
Wogonin-induced cellular senescence sensitized Navitoclax to kill T-cell malignancy cells. (a) Hut-102 and Jurkat cells were treated with or without 20 *μ*M wogonin for 5 d, and western blot was used to detect the expression change of BCL-2 and BCL-xL. GAPDH and *β*-actin were used as loading controls. (b) Relative protein expression levels of BCL-2 and BCL-xL. Columns represent the mean from three parallel experiments (mean ± SEM). ^∗^*p* < 0.05, ^∗∗^*p* < 0.01, compared with the control group. (c) Jurkat cells were exposed to 20 *μ*M wogonin for 5 d, and then, control or wogonin-treated Jurkat cells were treated with the indicated concentrations of Navitoclax (0-10 *μ*M) for 48 h. Cell viability was assayed by CCK8. The data represent the mean ± SEM of three different experiments. ^∗∗∗^*p* < 0.001, compared with the control group. (d) Jurkat cells were exposed to 20 *μ*M wogonin for 5 d, and then, control or wogonin-treated Jurkat cells were then treated with the indicated concentrations of Navitoclax (1.25, 5 *μ*M) for 48 h. Cell apoptosis was assayed by flow cytometry after Annexin-V/PI staining. Data represent the mean from three parallel experiments (mean ± SEM). ^∗^*p* < 0.05, ^∗∗^*p* < 0.01, and ^∗∗∗^*p* < 0.001, compared with the control group. (e) Jurkat cells were exposed to 20 *μ*M wogonin for 5 d, and then, control or wogonin-treated Jurkat cells were then treated with the indicated concentrations of Navitoclax (1.25 *μ*M) for 48 h. The protein expression levels of PARP-1, cleaved PARP-1, caspase-3, cleaved caspase-3, Bax, and Bim were analyzed by western blot. *β*-Actin were used as loading controls. (f) Relative protein expression levels of cleaved caspase-3/caspase-3, cleaved PARP-1/PARP-1, Bax, and Bim_L_. Columns represent the mean from three parallel experiments (mean ± SEM). ^∗^*p* < 0.05, ^∗∗^*p* < 0.01, and ^∗∗∗^*p* < 0.001, compared with the control group.

Recent research suggested that the expression of prosurvival networks increased in senescent cells including BCL-2, resulting in resistance to apoptosis [19]. We used senolytic drugs to target senescent cells, which is the second step of one-two punch therapy specific to Achilles' heel of senescent cells [19, 49]. Navitoclax, a senolytic BH3 mimetic drug, can selectively kill senescent cells [21, 50]. Navitoclax binds to BCL-2, BCL-xL, and BCL-w *in vitro*, while BCL-2 plays a key role *in vivo* and high expression of BCL-2 exhibited sensitivity to Navitoclax in human non-Hodgkin lymphomas instead of BCL-xL or BCL-w [51]. To investigate whether wogonin-induced senescence promoted the expression of prosurvival protein BCL-2 and BCL-xL, western blot was used and results showed that the expression of BCL-2 was upregulated but the expression of BCL-xL was not changed after treatment with 20 *μ*M wogonin for 5 d in Hut-102 and Jurkat cells (Figures 7(a) and 7(b)). We chose Jurkat cells to assess cell viability affected by Navitoclax. The CCK8 assay indicated that wogonin-induced senescent cells exhibited more significant sensitivity to Navitoclax than control cells (Figure 7(c)). Meanwhile, cell apoptosis analyses showed that Navitoclax could induce more apoptosis in wogonin-treated cells than in untreated cells (Figure 7(d)). Moreover, western blot results showed that the expression of apoptosis protein levels like cleaved caspase-3/caspase-3, cleaved PARP-1/PARP-1, Bax, and Bim_L_ was increased in Navitoclax-treated senescent cells (Figures 7(e) and 7(f)). In conclusion, wogonin-induced senescent cells were more sensitive to Navitoclax cytotoxicity and were effectively killed.

## 4. Discussion

T-cell malignancies remain to have poor prognosis and are difficult to have specific treatments [3, 52]. Clinically, chemotherapy based on the ABVD and CHOP regimen is still the preferred treatment for T-cell malignancies, and toxic effects and drug resistance brought by traditional chemotherapy regimens are unavoidable [53–55]. Cellular senescence makes senescent cells as a druggable target to repress tumor progression [22, 56]. Thus, inducing cellular senescence makes senescent cells display an induced vulnerability to senolytics drugs, which could be a novel one-two punch therapy for the treatment of T-cell malignancies [19, 50, 57].

*TP53* mutations in hematologic malignancies are less frequent (5%-15%) but closely associated with poor prognosis [58]. Different sites, forms, and frequencies of *TP53* mutation lead to different disease states [58–60]. Because of the central role of p53 in regulating cells' normal life activity, drug discovery concentrates on manipulating p53 and eradicating cancer cells. Wogonin is a well-known flavonoid and has a wide range of anticancer effects especially producing a better effect in hematologic malignancies [61–64]. However, although moderate and high concentrations of wogonin (over 40 *μ*M) had a better therapeutic effect, its insoluble characteristic and cytotoxicity in high doses were inescapable and limited its use in clinic. We found that low concentration of wogonin (20 *μ*M) could induce cellular senescence via DNA damage response in T-cell malignancies and verified the application of one-two punch therapy involving cellular senescence. Targeting senescent cells may benefit to increase therapeutic efficacy and reduce risk of recurrence [19]. Moreover, a previous study also reported that wogonin could target the Warburg Effect which depends on wild-type p53 to produce anticancer effects [65]. Except in the field of metabolism, p53 also plays an important role in regulating cellular senescence [66]. Our results showed that wogonin induced cellular senescence in p53 functional cell lines (Hut-102, Jurkat) but not the p53-deficient Hut-78 cell line. And we also found senescence-related SAHF phenomenon in Hut-102 and Jurkat cells but not in Hut-78 cells, suggesting that p53 may be involved in wogonin-induced senescence. Meanwhile, the phosphorylation of p53 (Ser15) was increased in senescent cells after the treatment with wogonin for 5 d. The knocking down assay confirmed that wogonin-induced senescence depended on the expression of p53. It is indicated that p53 may be a core factor in mechanisms of wogonin-treated T-cell malignancies.

According to our study, wogonin induced DNA damage response and increased the expression of *γ*-H2AX in Hut-102 and Jurkat cells. As the consequences of DNA damage response, phosphorylation of CHK2 (Thr68) was activated after the treatment with wogonin in senescent cells. The mRNA and protein expression of p21 playing a crucial role was obviously increased by wogonin treatment to render the cell cycle arrest irreversible and induce the senescence phenotype. The mRNA level of p27 did not change in Hut-102 and Jurkat cells, and the mRNA level of p16 had slightly changed in Jurkat cells but not in Hut-102 cells. It is also worth noting that the protein expression of p16 did not change in Hut-102 and Jurkat cells, and the results prompted us that the p16/Rb pathway may not mainly participate in wogonin-induced cellular senescence in T-cell malignancies. In a word, the results suggested that the CHK2/p53/p21 pathway may be involved in wogonin-induced cellular senescence.

DNA damage is only the mediator of cellular senescence, but the change of telomere function is one of the real causes to trigger DNA damage response resulting in cellular senescence [67]. Telomere dysfunction leads to aging, which is a proliferation disorder dependent on p53 and pRb, and hinders the continuous accumulation of mutations, thus resulting in blocking the development of cancer. In contrast, experimental inactivation of p53 and pRb enables fibroblasts to bypass aging and continue to divide [68, 69]. As a mild senescence inducer, nonlethal concentration of wogonin showed a unique function to T-cell malignancies. 20 *μ*M wogonin obviously suppressed the transcription of *C-MYC* and its downstream gene *hTERT*. Furthermore, wogonin decreased the protein expression of c-Myc and hTERT. *C-MYC* is a well-known oncogene and regulates the occurrence and progression of cancer. The promoter region of *hTERT* has oncoprotein c-Myc binding site-E-boxes, which induce the transcriptional activity of *hTERT* and activate telomerase [70]. We can infer that wogonin induced cellular senescence partly through the inhibition of telomere function. The underlying mechanisms are still being investigated.

In this paper, we used Hut-78 cells as the p53 deficiency model to verify the mechanism of wogonin. We did not observe obvious cellular senescence in Hut-78 cells after wogonin treatment. It is worth mentioning that we found that wogonin increased the level of *γ*-H2AX in Hut-78 cells (Figure S1a, b). Following the results, we detected the expression of CHK2, p-CHK2 (T68), p21, p16, and p27. The results showed that the expression of CHK2 and p-CHK2 (T68) were all increased after wogonin treatment for 7 d. The expression of p21 had slightly increased on day 7, but p16 and p27 had no change (Figure S1c, d). The upregulated folds of p21 in Hut-102 and Jurkat cells were more than those in Hut-78 cells. The expression of p21 was slightly increased in Hut-78 cells after treatment with wogonin slighter (Figure 6(d) and Figure S1d). However, the lack of p53 and slightly increased level of p21 may not be enough give rise to senescence in Hut-78 cells. CFSE experiments suggested that the decreased fluorescence intensity in CFSE-labeled cells was prevented by wogonin in Hut-78 cells. Thus, we supposed that there could be other signaling pathways to account for the mechanism which regulates cell proliferation induced by wogonin in Hut-78 cells. More follow-up studies are warranted to unveil the true nature of wogonin in Hut-78 cells.

Some studies have shown that overexpression of BCL-2 or BCL-xL is commonly observed in acute lymphoblastic leukemia (ALL) and chronic lymphocytic leukemia (CLL) [71, 72]. Navitoclax is a specific BH3-mimetic drug especially used in hematologic malignancies [73]. However, BCL-2 was verified as playing a key role in lymphoid cells, and overexpression of BCL-2 could increase the sensibility to Navitoclax [51]. But lymphoid cells with low expression of BCL-2 lack sensibility to Navitoclax and lose opportunity for optimal cell killing. The expression of BCL-2 was upregulated, contributing to the resistance of senescent cells to death. In our study, the increased expression of BCL-2 in senescent cells induced by wogonin could be detected by western blot. Then, the CCK8 assay showed that senescent cells were more sensitive to Navitoclax compared to untreated cells. Thus, the sequential combination of the BCL-2 inhibitor Navitoclax could promote the death of tumor cells, so there was no doubt that adopting this treatment method was of positive significance.

**Figure 8:**
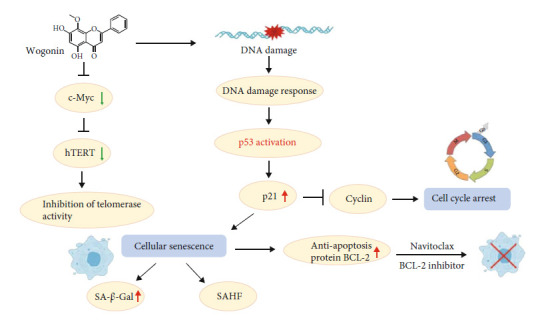
Graphical abstract on the mechanisms of one-two punch therapy involved in wogonin-induced cellular senescence in T-cell malignancies.

In summary, our findings suggest that wogonin not only inhibited telomere function but also induced p53-dependent cellular senescence, which was correlated with DDR. Cellular senescence promoted the expression of BCL-2 and consequently increased the sensibility to Navitoclax and removed senescent cells. Our work indicates prosenescence therapy induced by wogonin, sequentially combined with Navitoclax, providing a novel clinical approach for T-cell malignancy treatment (Figure 8).

## 5. Conclusion

In conclusion, our study revealed that wogonin inhibited telomerase activity via inhibition of c-Myc regulating the expression of hTERT. On this basis, we also found that wogonin induced p53-dependent cellular senescence mediated by DDR in T-cell malignancies. Moreover, wogonin-induced senescent cells increased the sensibility of Navitoclax resulting in killing T-cell malignancy cells effectively. cells. This may be considered a promising aspect of clinical cancer treatment.

## Data Availability

The authors declare that the data supporting the findings of this study are available from the corresponding author upon reasonable request.

## Additional Points

*Additional Information*. The online version of this article contains supplementary material, which is available to authorized users.

## Supplementary Materials

Supplementary Materials Supplemental Figure. Effect of 20 *μ*M wogonin on Hut-78 cell line. (a) Hut-78 cells were treated with or without 20 *μ*M wogonin for the indicated time periods (0, 1, 3, 5, 7 d), and western blot was used to detect the changes in the expression of DNA damage marker protein *γ*-H2AX. *β-a*ctin was used as loading controls. The right panel: relative protein expression level of *γ*-H2AX. Columns represent the mean from three parallel experiments (mean ± SEM). ^∗^*p* < 0.05, ^∗∗^*p* < 0.01, compared with control group. (b) Hut-78 cells were treated with or without 20 *μ*M wogonin for 7 d, then the cells were fixed, permeabilized and stained with antibody against *γ*-H2AX (Green), while nuclei were stained with DAPI (Blue). Immunofluorescent images showed the distribution of *γ*-H2AX in Hut-78 cells (original magnification ×1000; immersion objective ×100/×100 with immersion oil type). Images are representative of three independent experiments. (c) Hut-78 cells were treated with or without 20 *μ*M wogonin for the indicated time periods (0, 1, 3, 5, 7 d), and western blot was performed to detect the changes in the expression of CHK2, p-CHK2 (T68), p21, p16 and p27. *β*-actin was used as loading controls. (d) Relative protein levels of p-CHK2/CHK2, p16, p21 and p27 were determined. Columns represent the mean from three parallel experiments (mean ± SEM). ^∗^*p* < 0.05, ^∗∗^*p* < 0.01, compared with control group.
