# Supplementary figures and images for: One-Two Punch Therapy for the Treatment of T-Cell Malignancies Involving p53-Dependent Cellular Senescence
Source: Oxid Med Cell Longev. 2021 Sep 21;2021:5529518. doi: 10.1155/2021/5529518 (PMC8481056; doi:10.1155/2021/5529518)

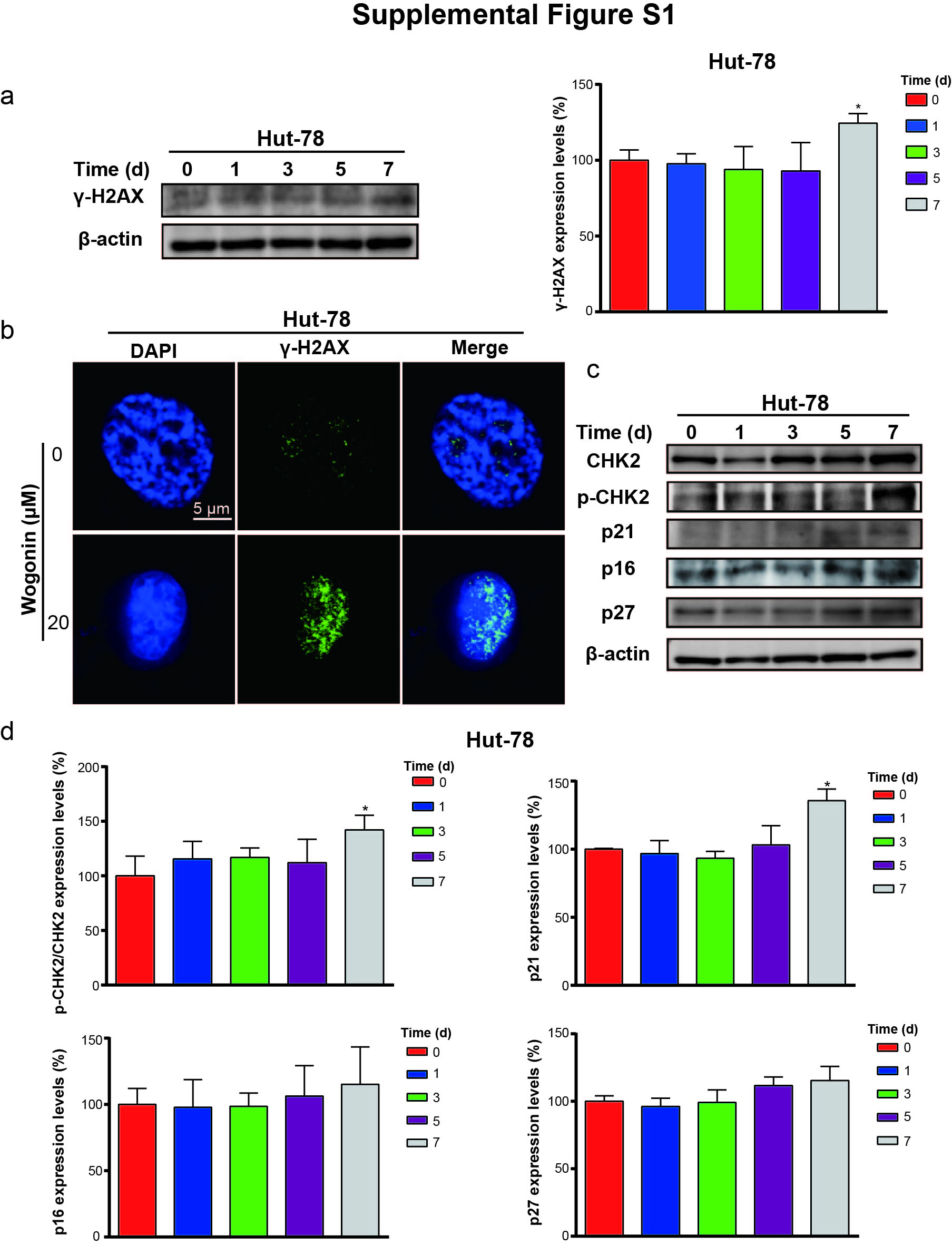

Supplement: Supplementary Materials — Supplemental Figure. Effect of 20 μM wogonin on Hut-78 cell line. (a) Hut-78 cells were treated with or without 20 μM wogonin for the indicated time periods (0, 1, 3, 5, 7 d), and western blot was used to detect the changes in the expression of DNA damage marker protein γ-H2AX. β-actin was used as loading controls. The right panel: relative protein expression level of γ-H2AX. Columns represent the mean from three parallel experiments (mean ± SEM). ∗p < 0.05, ∗∗p < 0.01, compared with control group. (b) Hut-78 cells were treated with or without 20 μM wogonin for 7 d, then the cells were fixed, permeabilized and stained with antibody against γ-H2AX (Green), while nuclei were stained with DAPI (Blue). Immunofluorescent images showed the distribution of γ-H2AX in Hut-78 cells (original magnification ×1000; immersion objective ×100/×100 with immersion oil type). Images are representative of three independent experiments. (c) Hut-78 cells were treated with or without 20 μM wogonin for the indicated time periods (0, 1, 3, 5, 7 d), and western blot was performed to detect the changes in the expression of CHK2, p-CHK2 (T68), p21, p16 and p27. β-actin was used as loading controls. (d) Relative protein levels of p-CHK2/CHK2, p16, p21 and p27 were determined. Columns represent the mean from three parallel experiments (mean ± SEM). ∗p < 0.05, ∗∗p < 0.01, compared with control group. [file 5529518.f1.jpg]
